# High prevalence of prescription of psychotropic drugs for older patients in a general hospital

**DOI:** 10.1186/s40360-017-0183-0

**Published:** 2017-12-04

**Authors:** Inken Arnold, Kati Straube, Wolfgang Himmel, Stephanie Heinemann, Vivien Weiss, Laura Heyden, Eva Hummers-Pradier, Roland Nau

**Affiliations:** 1 Department of Geriatrics, Evangelisches Krankenhaus Göttingen-Weende, An der Lutter 24, 37075 Göttingen, Germany; 2 Institute of Neuropathology, University Medical Center Göttingen, Robert-Koch-Str. 40, 37099 Göttingen, Germany; 3 Department of General Practice, University Medical Center Göttingen, Humboldtallee 38, 37073 Göttingen, Germany; 4 Department of Internal Medicine, Evangelisches Krankenhaus Göttingen-Weende, An der Lutter 24, 37075 Göttingen, Germany

**Keywords:** Pharmacoepidemiology, Drug use, Psychotropic drugs, Elderly, Retrospective study, Hospital

## Abstract

**Background:**

Many elderly patients receive psychotropic drugs. Treatment with psychotropic agents is associated with serious side effects including an increased risk of falls and fractures. Several psychotropic drugs are considered potentially inappropriate for treatment of the elderly.

**Methods:**

A retrospective chart review was conducted covering all patients aged ≥ 65 years who were admitted to Evangelisches Krankenhaus Göttingen-Weende between 01/01/2013 and 03/31/2013. Psychotropic drugs reviewed for included benzodiazepines, Z-drugs, antidepressants and neuroleptics, but not drugs for sedation during artificial ventilation or pre-medication before surgery. Potentially inappropriate drugs were identified according to the PRISCUS list. To assess which factors were associated with the administration of psychotropic drugs, univariate and multivariable logistic regression analyses were performed.

**Results:**

The charts of 2130 patients (1231 women) were analyzed. 53.9% of all patients received at least one psychotropic medication (29.5% benzodiazepines, 12.6% Z-drugs, 22.2% antidepressants, 11.9% neuroleptics). The mean number of psychotropic drugs prescribed per patient with at least one prescription was 1.6. Patients treated in the geriatric department most often received antidepressants (45.0%), neuroleptics (20.6%) and Z-drugs (27.5%). Benzodiazepines and Z-drugs were prescribed mostly as medication on demand (77.7% of benzodiazepines, 73.9% of Z-drugs). Surgical patients most frequently received benzodiazepines (37.1%). Nearly one-third of all patients ≥ 65 years was treated with at least one potentially inappropriate psychotropic medication. The mean number of potentially inappropriate psychotropic medications per patient with at least one psychotropic prescription was 0.69. The percentage of patients with potentially inappropriate psychotropic medication was highest in the surgical departments (74.1%). Female gender (adjusted OR 1.36; 95% CI 1.14 to 1.63), stay in the Department of Geriatrics (2.69; 2.01 to 3.60) or the interdisciplinary intensive care unit (1.87; 1.33 to 2.64) and age ≥ 85 years (1.33; 1.10 to 1.60) were associated with psychotropic drug treatment.

**Conclusions:**

A high percentage of patients aged ≥ 65 years received psychotropic drugs. The chance that a potentially inappropriate psychotropic drug would be administered was highest in the surgical departments. Antidepressants, neuroleptics and Z-drugs were used surprisingly often in geriatric medicine. Educational strategies could reduce the use of psychotropic drugs and the prescription of potentially inappropriate medications.

## Background

A high percentage of older patients are administered psychotropic drugs, despite the considerable risks associated with their use. Falls, drug-induced cognitive decline and excess mortality during treatment with psychoactive drugs [1–8] are frequent problems in the care of older people. Especially benzodiazepines and Z-drugs are frequently prescribed to older persons with sleeping problems. Medication-related fall risk factors have been identified in previous studies [9]. The risk of falls is evident for psychotropic drugs, but also for antihypertensives and polymedication [4, 10, 11]. The Swedish National Board of Health and Welfare (NBHW) has generated a list of fall-risk increasing drugs (FRID) which includes opioids, neuroleptics, anxiolytics, hypnotics and sedatives, and antidepressants [12].

Benzodiazepines, Z-drugs, antidepressants and neuroleptics contribute to cognitive deficits in patients [13–15]. Chronic use of benzodiazepines is associated with a lower latent cognitive level [16]. Antidepressants or neuroleptics with strong anticholinergic side effects are more likely to induce cognitive decline than less anticholinergic drugs [17]. Excess mortality due to the use of psychoactive drugs has been extensively studied with neuroleptics. Up to one-third of nursing home residents receive at least one neuroleptic drug [17, 18]. Both conventional and atypical neuroleptic drugs increase mortality in the elderly by a factor of approx. 1.5–1.7 [3, 7]. The risk increases with higher dosages and is highest in the first weeks after start of therapy [1, 8]. Moreover, the risk of cerebrovascular events appears to be elevated by 1.3 to 2 in older people taking neuroleptics [19, 20].

In view of the severe potential side effects of psychotropic drugs and the potential for improvement by adhering to guidelines for their use, we conducted a retrospective survey of the current practice in patients older than 65 years. We hypothesized that in some departments sleep disorders very frequently were treated with benzodiazepines, and that not all departments adhered to the PRISCUS list [21], an expert opinion-based list of potentially inappropriate drugs in the elderly that is currently used as a guideline in Germany. Therefore, we assessed the frequencies and average daily doses of treatments with benzodiazepines, Z-drugs, antidepressants and neuroleptics in patients ≥65 years in relation to the department, in which the patient was treated, and to the recommendations of the PRISCUS list. The study took place in a regional general hospital with several surgical departments as well as departments of general internal and geriatric medicine.

## Methods

### Sampling

The study was carried out in a regional hospital in Lower Saxony, Germany, which focusses on basic and standard in-patient care. There are seven departments with a total of 485 beds: internal medicine, geriatrics (acute geriatrics and geriatric rehabilitation), trauma surgery/orthopedics, general surgery, plastic surgery, urology and oto-rhino-laryngology. We retrospectively reviewed the paper hospital charts of all patients aged 65 years or older who were admitted to the hospital for at least one night in the time period between 01/01/2013 to 03/31/2013. The period in the past was chosen to ensure that most of the hospital charts were accessible in the hospital archive and not in current use for administrative purposes. In 2016, we began interventions to reduce the consumption of benzodiazepines. We expect that the prescriptions of psychotropic drugs between 2014 and 2016 did not differ substantially from 2013; the effect of the interventions will become apparent in the course of 2017 and 2018. Patients who died were not included in the study, because they often received benzodiazepines in their last days of life as part of their palliative care. Furthermore, psychotropic drugs applied as sedatives for artificial ventilation or pre-medication before surgery or in the postanaesthesia care unit were not taken into consideration including those used for agitation after surgery, i.e. at the end of sedation.

### Data collection

Patients’ age, sex, department and duration of stay were retrieved from computerized lists of patients available for the whole hospital. These lists were used to order the paper charts from the archive. The patients’ data including diagnoses were gathered from the charts and documented anonymously using a computer-based standardized case report form (CRF). Possible predisposing factors for a treatment with psychotropic drugs were also noted: living place before and after the present hospital stay (own home, nursing home, other department in same hospital, other hospital or unknown), medication or drug addictions (if documented). Intra-hospital transferees (161 patients, 7.6%) were counted as one patient in each department. This was necessary due to the fact that after each intra-hospital transfer, a new prescription plan was set up under the guidance of the physician or surgeon in charge of the patient. Otherwise we would have been unable to calculate department-specific differences.

The use of benzodiazepines, Z-drugs, antidepressants and antipsychotic or sedative neuroleptics during the hospital stay was recorded in detail: the name of the drug, the number of days the drug was administered, and based on this information the average daily dose. Furthermore, we documented whether drug treatments were initiated or stopped or whether there was any attempt to reduce the dosage during the hospital stay. Besides data concerning the hospital period, we also recorded the psychotropic drug medication before and after hospitalization (as documented in the admission form and the discharge letter). “Discontinued medication” referred exclusively to the drugs a patient had been receiving prior to hospital admission that were stopped during hospital stay. The study was approved by the Ethics Committee of the University Medical Center Göttingen (reference number: 25/2/14).

### Statistical analysis

To facilitate data handling and comparisons, several variables were grouped into classes. The departments were categorized into three groups: surgical departments (trauma surgery/orthopedics, general surgery, plastic surgery, urology and oto-rhino-laryngology), general internal medicine and geriatrics. This categorization was based on the different attributes of the departments, which were comparable in all surgery departments (similar intensity of doctor-patient contact, similar drug management procedures, similar processes in admission and discharge) as opposed to the Departments of Internal Medicine (more drug-centered treatment) and Geriatrics (high incidence of depression and dementia, longer time of stay, rehabilitative approach, interest of the staff not only in medical, but also in social conditions, special focus on the medication regime, older patients). This categorization of the departments allowed departments with only few patients (e.g. oto-rhino-laryngology, *n* = 2) to be included in the analysis. Patient age was divided into high age (65-84 years) and very high age (≥ 85 years) for the multivariable logistic regression analysis.

The dependence of patient characteristics and the drugs prescribed on the department category are described in absolute and relative numbers. Furthermore, the prescription frequency of the different psychotropic substances and differences in prescribing between the departments were of main interest. To assess which factors were significantly associated with the administration of at least one psychotropic substance, we performed univariate and multivariable logistic regression analyses and calculated the odds ratios (ORs) and their corresponding 95% confidence intervals (CIs) as measures of effect size [22]. Goodness of fit of the multivariable analysis was assessed by the Hosmer-Lemeshow test, with *p*-values > 0.05 supporting the model’s adequacy.

### Potentially inappropriate drugs according to the PRISCUS-list

To identify potentially inappropriate drugs for the elderly, we based our analysis on the German PRISCUS list [21]. This list names substances associated with a high risk of side effects in patients aged ≥ 65 years. For some compounds, the list defines a maximum recommended daily dose, others are classified as potentially inappropriate regardless of dosage. The basis for the classification as potentially inappropriate was either the administration of the drug at any dose (if the drug was considered potentially inappropriate irrespective of its dose) or the average daily dose determined by the questionnaire (if the drug was considered potentially inappropriate only at high doses). Potentially inappropriate psychotropic drugs according to the PRISCUS list which were used in patients treated at our institution were: benzodiazepines - lormetazepam > 0.5 mg/d, lorazepam > 2 mg/d, brotizolam > 0.125 mg/d, oxazepam > 60 mg/d, diazepam, temazepam, bromazepam, dipotassium clorazepat, flunitrazepam, alprazolam, clonazepam, nitrazepam, flurazepam; Z-drugs - zopiclone > 3.75 mg/d, zolpidem > 5 mg/d; antidepressants - amitriptylin, doxepine, trimipramine, clomipramine, imipramine, maprotiline, fluoxetine; neuroleptics - haloperidol > 2 mg/d, olanzapine > 10 mg/d, levomepromazine, clozapine.

## Results

### Sample

A total number of 2130 patients aged 65 years or older were included, 57.8% (*n* = 1231) of the patients were women, 42.2% (*n* = 899) men.

The mean age was 79.2 years, and the mean length of stay was 9.1 days. The mean length of stay ranged from 7 days at the Dept. of Internal Medicine and the surgical departments to 23 days at the Dept. of Geriatrics. The longest hospital stay of an individual patient was 72 days. Most patients admitted to the hospital came from their own homes (65.2%). 12.1% came to the hospital from nursing homes, 7.6% from another department of the same hospital, 6.4% from another hospital, and in 8.7% of the patients the previous residence was not documented (most of these patients came probably from their own homes). Demographic and clinical characteristics are presented in Table 1. Two hundred thirty patients (10,8%) had a diagnosis of dementia documented in the discharge letter, and in 124 (5,8%) depression was diagnosed.

**Table 1:** Age distribution of the sample

					Age			
		n	%	mean	SD	median	min	max
Sex	Department							
Female	surgical departments	512	24.0	78.3	7.8	77	65	100
	internal medicine	525	24.7	80.9	7.6	82	65	101
	geriatrics	194	9.1	83.8	6.7	85	67	99
	total	1231	57.8	80.3	7.8	80	65	101
Male	surgical departments	438	20.6	75.7	6.7	75	65	95
	internal medicine	364	17.1	78.7	7.1	79	65	97
	geriatrics	97	4.6	82.4	6.3	83	69	95
	total	899	42.2	77.6	7.2	77	65	97
Department								
surgical depts.		950	44.6	77.1	7.4	76	65	100
internal medicine		889	41.7	80	7.5	80	65	101
geriatrics		291	13.7	83.3	6.6	85	67	99
total		2130	100	79.2	7.7	79	65	101

Age in years, SD = standard deviation, surgical departments include: trauma/orthopedic surgery, urology, otorhinolaryngology, general surgery and plastic surgery

### Prevalence of in-hospital consumption of psychotropic drugs

**Fig. 1:**
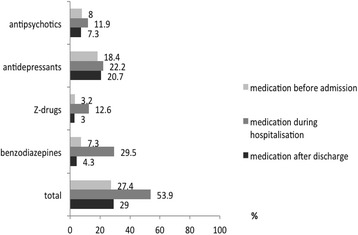
Prevalence of the prescription of psychotropic drugs (*N* = 2130) before admission, during hospital stay and after discharge in a German general hospital

Approximately half of the patients (53.9%) received at least one psychotropic drug during their hospital stay, with benzodiazepines being the most frequently prescribed psychotropic drugs. 27.4% of the patients received a psychotropic medication before admission and 29.0% after discharge (Fig. 1). Patients who received at least one psychotropic drug in the hospital (*n* = 1149) had a longer hospital stay than those who did not receive any psychotropic drug (*n* = 981) (10.95 ± 10.07 days versus 6.93 ± 6.90 days, *p* < 0.0001, t-test). The mean number of psychotropic drugs prescribed per patient in patients who received at least one prescription of a psychotropic drug was 1.6.

In hospital, antidepressants and neuroleptics with sedative effects were prescribed more often than stimulating or antipsychotic agents; 14.2% vs. 11.7% for antidepressants and 8.7% vs. 4.9% for neuroleptics. The most frequently prescribed psychotropic drugs were: lormetazepam, lorazepam, zopiclone, mirtazapine, citalopram and melperone (Table 2).

**Table 2:** Prevalence of all psychotropic drugs prescribed in the period studied

Psychotropic drugs	n	%	Potentially inappropriate (%)
benzodiazepines			
lormetazepam^a^	403	18.9	> 0,5 mg/d (97.3)
lorazepam^a^	155	7.3	> 2 mg/d (1.9)
tetrazepam	26	1.2	each dose (100)
diazepam	25	1.2	each dose (100)
oxazepam	25	1.2	> 60 mg/d (0)
temazepam	15	0.7	each dose (100)
bromazepam	12	0.6	each dose (100)
dipotassium clorazepat	10	0.5	each dose (100)
flunitrazepam	9	0.4	each dose (100)
alprazolam	5	0.2	each dose (100)
brotizolam	5	0.2	> 0.125 (80)
clonazepam	3	0.1	each dose (100)
midazolam	3	0.1	n.a.
nitrazepam	2	0.1	each (100)
flurazepam	1	0.1	each dose (100)
neuroleptics			
melperone	119	5.6	n.a.
prothipendyl	58	2.7	n.a.
quetiapine	42	2.0	n.a.
haloperidol	33	1.6	> 2 mg/d (42.4)
promethazine	29	1.4	n.a.
risperidone	20	0.9	n.a.
aripiprazole	6	0.3	n.a.
olanzapine	6	0.3	> 10 mg/d (50)
pipamperone	3	0.1	n.a.
levomepromazine	2	0.1	each dose (100)
clozapine	2	0.1	each dose (100)
flupentixol	1	0.1	n.a.
pimozid	1	0.1	n.a.
sulpirid	1	0.1	n.a.
Z-drugs			
zopiclone	171	8.0	> 3,75 mg/d (60.8)
zolpidem	112	5.3	> 5 mg/d (76.8)
antidepressants			
mirtazapine	228	10.7	n.a.
citalopram	138	6.5	n.a.
duloxetine	47	2.2	n.a.
venlafaxine	34	1.6	n.a.
amitriptyline	31	1.5	each dose (100)
doxepine	22	1.0	each dose (100)
sertraline	14	0.7	n.a.
trimipramine	12	0.6	each dose (100)
escitalopram	10	0.5	n.a.
opipramol	9	0.4	n.a.
trazodone	5	0.2	n.a.
maprotiline	5	0.2	each dose (100)
fluoxetine	5	0.2	each dose (100)
paroxetine	5	0.2	n.a.
clomipramine	4	0.2	each dose (100)
bupropion	4	0.2	n.a.
agomelatin	2	0.1	n.a.
mianserin	2	0.1	n.a.
imipramine	2	0.1	each dose (100)
fluvoxamine	1	0.1	n.a.

% of potentially inappropriate refers to patients receiving the respective psychotropic drug

^a^Average daily doses [mg] of the psychotropic drugs most frequently prescribed (medians). Lormetazepam: 1.0; lorazepam: 1.0; zopiclone 7.5; zolpidem 10.0; mirtazapine: 15; citalopram: 20; melperone: 25; prothipendyl: 50

Benzodiazepines and Z-drugs were prescribed mostly as medication on demand (77.7% of benzodiazepines, 73.9% of Z-drugs). Neuroleptics were prescribed both as long-term medication (60.1%) and medication on demand (39.9%), whereas antidepressants were prescribed almost exclusively as long-term medication (99.6%).

**Fig. 2:**
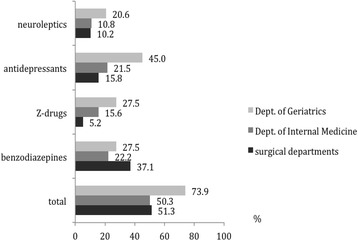
Percentage of patients (*N* = 2130) receiving psychotropic drugs analyzed by department in a German general hospital

Compared to patients treated in the surgical departments (51.3%) and the Dept. of Internal Medicine (50.3%), patients in the Dept. of Geriatrics were more likely to receive psychotropic drugs (73.9%). All groups of psychotropic drugs were prescribed most frequently to patients in the Dept. of Geriatrics with the exception of benzodiazepines, which were prescribed most frequently to patients in one of the surgical departments (Fig. 2).

In 9.3% of all patients ≥ 65 years at least one new psychotropic drug was started during their hospital stay and recommended in the discharge letter (Table 3). The most frequent newly started psychoactive compounds were the antidepressants mirtazapine (*n* = 81, 3.8%) followed by citalopram (*n* = 20, 0.9%). Melperone (*n* = 24, 1.1%), tetrazepam (*n* = 17, 0.8%) and zolpidem (*n* = 10, 0.5%) were the most frequent newly prescribed neuroleptic, benzodiazepine and Z-drug. In 5.5% of patients a long-term psychotropic medication that the patients had been receiving prior to hospital admission was discontinued. Tricyclic antidepressants (amitriptyline, doxepine, trimipramine, nortriptyline; *n* = 22), citalopram (*n* = 20) and mirtazapine (*n* = 13) were the most frequently discontinued antidepressants, melperone (*n* = 11), risperidone (*n* = 7) and promethazine (*n* = 5) were the most frequently discontinued neuroleptics. The most frequently discontinued benzodiazepines and Z-drugs were zopiclone (*n* = 8, 0.4%), lorazepam (*n* = 6, 0.3%) and lormetazepam (*n* = 6, 0.3%). Overall, geriatricians prescribed a new treatment with psychotropic drugs most frequently, but also most often discontinued other long-term psychotropic medications.

**Table 3:** Number of patients receiving a new prescription of psychotropic drugs or a discontinuation of long-term psychotropic medication

	Total (*N* = 2130)	Benzodiazepines	Z-drugs	Antidepressants	Neuroleptics
new prescription	198 (9.3%)	33 (1.6%)	17 (0.8%)	126 (5.9%)	41 (1.9%)
long-term medication discontinued	118 (5.5%)	27 (1.3%)	15 (0.7%)	61 (2.9%)	31 (1.5%)

New prescription refers to psychotropic drugs newly prescribed during hospital stay and recommended in the discharge letter, long-term medication discontinued refers to medication, which was prescribed prior to hospital admission and discontinued during the hospital stay

### Factors associated with the prescription of psychotropic drugs

Three factors were significantly predictive in the univariate model for the prescription of at least one psychotropic drug: staying on an intensive care unit or the geriatric department, female gende and high age (Table 4). Treatment in the intensive care unit and in the Dept. of Geriatrics and gender remained statistically significant predictors of treatment with psychotropic drugs in the multivariable model (goodness of fit by Hosmer-Lemeshow test: *p* = 0.16), with a stay in the geriatric department being the strongest predictor (OR: 2.69; 95% CI: 2.01–3.60). In this multivariable analysis, high age (≥ 85 years) failed to be a significant factor for the prescription of a psychotropic drug.

**Table 4:** Factors associated with the prescription of psychotropic drugs

Influencing variables		Univariate model			Multivariable model^a^		
	%	OR	95%CI	*p* value	OR	95%-CI	*p* value
intensive care unit^b^							
no	52.8	1.0					
yes	67.7	1.87	(1.33–2.64)	0.0004	2.08	(1.47–2.96)	0.0001
sex							
male	48.8	1.0					
female	57.7	1.43	(1.20–1.70)	0.0001	1.36	(1.14–1.63)	0.0006
age							
65–84 years	51.9	1.0					
≥ 85 years	58.9	1.33	(1.10–1.60)	0.0036	1.10	(0.90–1.35)	0.33
department							
surgical departments	51.3	1.0					
internal medicine	50.3	0.96	(0.80–1.15)	0.67	0.96	(0.80–1.16)	0.70
geriatrics	73.9	2.69	(2.01–3.60)	0.0001	2.68	(1.99–3.61)	0.0001

*P* values, odds ratios and confidence intervals from univariate and multivariable logistic regression. (%) refers to patients prescribed psychotropic drugs based on *N* = 2130, OR odds ratio, CI confidence interval

^a^Goodness of fit (Hosmer–Lemeshow test: *p* = 0.16)

^b^Patients were treated at the intensive care unit (ICU) for part of their hospital stay. Psychotropic drugs administered during the ICU stay were not included in this evaluation

We repeated the multivariable analysis for benzodiazepines and Z-drugs together (goodness of fit by Hosmer-Lemeshow test: *p* = 0.80). The probability that a patient would receive a benzodiazepine or Z-drug was higher in the Dept. of Geriatrics compared to the surgical departments (OR 1.5; 95% CI 1.14–1.97; *p* = 0.004). This was a consequence of the relatively frequent use of Z-drugs in the Dept. of Geriatrics. Age was inversely correlated with the prescription of benzodiazepines and Z-drugs, i.e., patients ≥ 85 years were less likely to receive these drugs than patients between 65 and 84 years (OR 0.79; 95% CI 0.65–0.97; *p* = 0.023). The multivariate analysis was also repeated with potentially inappropriate psychotropic drugs as dependent variable (goodness of fit by Hosmer-Lemeshow test: *p* = 0.50). Treatment in one of the surgical departments was associated with an increased probability of receiving a potentially inappropriate psychotropic medication compared with treatment in the Dept. of Geriatrics (OR 1.68; 95% CI 1.23–2.29; *p* = 0.001). Age < 85 years was associated with the prescription of potentially inappropriate psychotropics, i.e., younger patients were more likely to receive these drugs than older ones (OR 1.89; 95% CI 1.50–2.36; *p* < 0.0001).

### Dementia and the prescription of neuroleptics

Of 230 patients with a diagnosis of dementia, 105 (45.7%) received at least one neuroleptic drug during their hospital stay. Eighty-two patients received one neuroleptic, 14 patients two different neuroleptics and 9 patients three neuroleptics. The diagnosis dementia was strongly associated with the prescription of at least one neuroleptic drug (*p* < 0.0001, Fisher’s exact test). There was no association between the prescription of an anti-dementia drug and a neuroleptic (*p* = 0.73, Fisher’s exact test).

### Potentially inappropriate psychotropic drugs

Nearly one-third of all elderly patients was treated with at least one potentially inappropriate psychotropic medication or an inappropriately high dosage. More than half of those who were treated with psychotropic drugs received at least one potentially inappropriate psychotropic medication (58.5%).

**Fig. 3:**
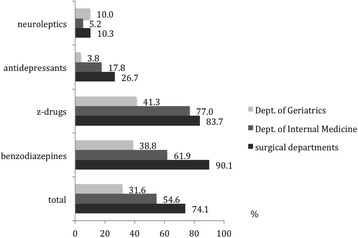
Percentage of patients receiving at least one potentially inappropriate psychotropic drug analyzed by department. Patients with at least one potentially inappropriate psychotropic drug are given as a percentage of all patients receiving psychotropic drugs. For the definition of potentially inappropriate drugs see Methods and the PRISCUS list [22]

Differences were observed between the individual departments. Compared to the Dept. of Geriatrics (31.6%) and Internal Medicine (54.6%), patients treated in one of the surgical departments received the highest proportion of potentially inappropriate psychotropic drugs (74.1% of all patients receiving psychotropic drugs). The use of benzodiazepines at inappropriate doses in the surgical departments was most prominent (90.1%) (Fig. 3). The mean number of psychotropic drug prescriptions per patient was 0.88 (0.78 in the surgical departments, 0.80 in the Dept. of Internal Medicine, 1.46 in the Dept. of Geriatrics). Overall, the mean number of potentially inappropriate psychotropic medication per patient with at least one psychotropic drug prescription was 0.69. It was highest in patients treated in the surgical departments (0.90), intermediate in patients treated in the Dept. of Internal Medicine (0.63) and lowest in patients treated in the Dept. of Geriatrics (0.36). Most patients only received one potentially inappropriate psychotropic drug or an inappropriate dose during their hospital stay, but some patients received different potentially inappropriate psychotropic drugs, one patient was even prescribed 6 different potentially inappropriate psychotropic drugs during his hospital stay (Table 5).

**Table 5:** Proportion of patients with 0 to 7 psychotropic drugs/ potentially inappropriate psychotropic medication during hospital stay

	Surgical Depts.	Dept. of Internal Medicine	Dept. of Geriatrics	Total (*N* = 2130)
all psychotropic drugs				
0	463	442	76	981
1	328	284	95	707
2	96	96	62	254
3	42	44	34	120
4	12	11	18	41
5	6	9	3	18
6	1	3	3	7
7	2	0	0	2
potentially inappropriate psychotropic drugs				
0	589	645	223	1457
1	302	212	59	573
2	45	29	9	83
3	11	1	0	12
4	2	2	0	4
5	0	0	0	0
6	1	0	0	1

For the definition of potentially inappropriate psychotropic medication see Methods and Table 2

The most frequent potentially inappropriate prescriptions according to the PRISCUS list were high doses of lormetazepam (> 0,5 mg/d, *n* = 392, mostly 1 mg/d), zopiclone (> 3,75 mg/d, *n* = 104, mostly 7.5 mg/d), zolpidem (> 5 mg/d, *n* = 86, mostly 10 mg/d), haloperidol (> 2 mg/d, *n* = 14, 2.5 mg/d or higher) and tricyclic antidepressants (amtriptyline *n* = 31, doxepine *n* = 22, trimipramine *n* = 12, clomipramine *n* = 4, imipramine *n* = 2) at any dose.

## Discussion

The proportion of patients ≥ 65 years receiving psychotropic drugs in this hospital was high, i.e., 53.9% of the patients received at least one psychotropic drug during their hospital stay. With interdepartmental variations, this applied to benzodiazepines, Z-drugs, antidepressants and neuroleptic drugs. Especially in the surgical departments, we detected a high proportion of patients treated with potentially inappropriate psychotropic drugs.

In general, the proportion of patients ≥ 65 years treated with psychotropic drugs was highest in the Dept. of Geriatrics. Several reasons may account for this: 1. Many geriatric patients suffered from geriatric multimorbidity including depression, chronic pain, insomnia, but also agitation during the day or night, hallucinations and delusions. 2. As recommended by the World Health Organization [23], antidepressants were used as adjuvants in analgesic medications at our institution. In a retrospective study, it often is difficult to identify the reason(s), why a drug was started. Often, more than one indication was present for the initiation of therapy, e.g., depression, sleep disturbance and weight loss as the reasons to choose mirtazapine. In the present study, therefore, no attempt was made to evaluate the indication for the prescription of an antidepressant. 3. In part, the antidepressant mirtazapine and the neuroleptics melperone and pipamperone were already used in the Dept. of Geriatrics during the study period as substitutes for benzodiazepines and Z-drugs for sleep induction. 4. The mean hospital stay of geriatric patients was longer than the mean stay in the other departments (23 days versus 7 days in the Dept. of Internal Medicine and in the surgical departments). One reason could be that the chance to develop symptoms necessitating treatment with a psychotropic drug was increased in geriatrics. Alternatively, patients requiring psychotropic drugs needed longer hospital stays to recover than patients who did not need psychotropics. Indeed, in the present study the duration of the hospital stay was longer in patients receiving psychotropic drugs than in those who did not receive any psychotropic medication (*p* < 0.0001).

Our data compare well with recently published data from nursing homes [5, 24, 25]. Residents in nursing homes, particularly those with dementia, often receive psychotropic drugs, among these even a high percentage of neuroleptics which may shorten their lives [1–4, 6–8]. In a recent Swedish study that reviewed all persons living in geriatric care units in Northern Sweden, in 2007 25.4% were reported to have received antipsychotic drugs, and 35.5% anxiolytic, hypnotic, or sedative drugs [24]. In Scotland, 28.4% of the population ≥ 65 years living in a care home received a benzodiazepine or a Z-drug [25].

When the medication prior to admission and the recommendations at discharge were compared (Table 3), 9.3% of all patients studied received a psychotropic drug as a new prescription, and in 5.5% a long-term medication was discontinued, i.e. the difference was 3.8%. Three percent of this difference was caused by antidepressants (5.9% of patients with prescription during hospital stay and recommendation in the discharge letter, 2.9% of patients whose antidepressants were stopped during their hospital stay). Conversely, new long-term prescriptions and discontinuation of long-term medication of benzodiazepines, Z-drugs and neuroleptics were almost equal. Hence, the high benzodiazepine and Z-drug use in in-patients did not result in an increase of the prescription of these drugs at discharge. This might be a result of the fact that the in-house medication of these drugs mostly was a medication on demand, which often was not included in the discharge letter.

In recent years, efforts to reduce the consumption of psychotropic drugs in institutions caring for older people have shown some success. The above mentioned Swedish study on patients living in geriatric care units noted that in 2013 the use of antipsychotic drugs had declined to 18.9%, and the prescription of anxiolytic, hypnotic, and sedative drugs had fallen to 29.4% [24]. Similarly, a mild decrease in the benzodiazepine use was observed in France between 2006 and 2012 in a population-based study [26]. Conversely, the prevalence of people receiving antidepressant drugs remained unchanged at approx. 50%. In the Swedish study, the use of anti-dementia drugs rose from 17.9 to 21.5% [24]. Anti-dementia drugs are often prescribed in an attempt to reduce the use of neuroleptics. In the present study, the prescription of an anti-dementia drug and a neuroleptic were not inversely associated, i.e., we found no evidence that the prescription of anti-dementia medication was associated with a reduced use of neuroleptics. Recently, it was suggested that physicians prescribing benzodiazepines to their elderly patients should educate these patients about the risks of benzodiazepine use and, when appropriate, offer them tapering protocols. A substantial proportion of long-term users appears to be able to taper off benzodiazepines via low-intensity interventions [27]. The present study was part of a project aiming at the reduction in the consumption of benzodiazepines and Z-drugs in our hospital [28] by developing in-house recommendations for the pharmacological treatment as well as education about alternative options for sleep problems.

The wide use of potentially inappropriate psychotropic medications in all departments was striking. The most frequent reason for this was surpassing the recommended daily doses of drugs considered potentially inappropriate at high doses. One probable reason is that many medical doctors are not familiar with lists of potentially inappropriate drugs for older patients. Another important reason appears to be the wish of the patient to increase the dose of a benzodiazepine or Z-drug above the doses considered appropriate for the elderly [22]. The prescription of potentially inappropriate psychotropic medication was most frequent in the surgical departments. There is evidence that avoiding potentially inadequate medication in the elderly reduces complications, particularly cognitive impairment, falls and all-cause mortality [4, 9, 29–32]. Adherence to the PRISCUS list has been one goal of the interventions started in 2016 in the hospital studied here.

The main strength of this study is the extensive and detailed assessment of all psychotropic medication administered to the entire elderly patient population of a German general hospital in a particular time period. The medications were classified according to the widely acknowledged PRISCUS list [22], an adaptation of Beer’s criteria and their revisions [33, 34] to German conditions, which helped detect shortcomings and a potentially inappropriate or even dangerous use of these drugs. In several instances, the maximum recommended dose according to the PRISCUS list is lower than the maximum dose allowed in the officially approved product label.

One weakness of our study is the assessment of the year 2013. It is known that interventions can reduce the consumption of benzodiazepines in hospitals. Since interventions to reduce the consumption of benzodiazepines were started in 2016, we expect that in the period 2014-2016 the prescriptions of psychotropic drugs were very similar to 2013, and that the interventions of 2016 will begin to show effects in 2017 and 2018. It may be considered a weakness of this study that intra-hospital transferees were counted as one patient in each department. If we had not done this, however, we would not have been able to calculate department-specific differences. The main weakness of our study was the inability to identify the real cause of the prescription of a psychotropic drug. This was a consequence of several shortcomings: 1. We conducted a retrospective study, and the daily comments of the medical doctors in the patients’ charts often were not detailed enough to identify the indications for the use of a psychotropic drug. 2. Often, one drug was prescribed for more than one indication (e.g., duloxetine or mirtazapine for depression and as analgesic adjuvants, quetiapine as an antipsychotic and as sleep inductor in patients with Parkinson’s disease). 3. Approximately 11% of the patients analyzed in this study suffered from dementia. Patients with dementia often express discomfort, but frequently do not report their complaints in detail, which complicates the diagnosis of pain or depression and may result in an overuse of psychotropic drugs.

## Conclusion

In a German general hospital, psychotropic drugs were frequently prescribed to patients ≥ 65 years, and a high percentage of older patients received potentially inappropriate psychotropic medication. Whereas in the Dept. of Geriatrics the proportion of patients receiving psychotropic drugs was highest, the proportion of patients receiving potentially inappropriate psychotropics was highest in the surgical departments. Awareness and the development of hospital-tailored guidelines for the treatment of sleep disturbances, delirium, dementia and depression may reduce the frequency of the use of psychotropic medication in general, and of potentially inappropriate psychotropic drugs or doses in particular.

## Abbreviations

CI: Confidence interval
CRF: Case report form
FRID: Fall-risk increasing drugs
NBHW: Swedish National Board of Health and Welfare
OR: Odds ratio
p: Probability
PRISCUS: Literal translation: venerable; Project “Prerequisites for a New Health Care Model for Elderly People with Multi-Morbidity”, funded by the German Ministry of Education and Research (BMBF)
Z-drugs: A group of non-benzodiazepine drugs with effects similar to benzodiazepines, whose names start with the letter “Z”
